# Involvement of a tick-borne orthomyxovirus matrix protein in vRNP nuclear export

**DOI:** 10.1128/jvi.01494-25

**Published:** 2025-12-02

**Authors:** Vaille A. Swenson, Jack Hemsath, Iris Yousaf, Carla Weisend, Michael A. Barry, Hideki Ebihara, Satoko Yamaoka

**Affiliations:** 1 Mayo Clinic Graduate School of Biomedical Sciences 32864 , Rochester, Minnesota, USA; 2 Kansas City University College of Osteopathic Medicine, Farber McIntire Campus, Joplin, Missouri, USA; 3 Department of Molecular Medicine, Mayo Clinic 6915, Rochester, Minnesota, USA; 4 Division of Infectious Diseases, Department of Medicine, Mayo Clinic 6915, Rochester, Minnesota, USA; 5 Department of Immunology, Mayo Clinic 6915, Rochester, Minnesota, USA; 6 Department of Virology 1, National Institute of Infectious Diseases, Tokyo, Japan; Cornell University Baker Institute for Animal Health, Ithaca, New York, USA

**Keywords:** Dhori virus, *Thogotovirus*, nuclear export protein, nuclear export, CRM1, vRNP, orthomyxovirus

## Abstract

Thogotoviruses are arthropod-borne viruses belonging to the genus *Thogotovirus* within the family *Orthomyxoviridae*. Like other orthomyxoviruses, such as the influenza A viruses (IAV), thogotoviruses replicate in the nucleus. As a result, progeny viral ribonucleoprotein complexes (vRNPs) must be exported to the cytoplasm prior to virion assembly and budding at the plasma membrane. In IAV, this export depends on binding of the viral nuclear export protein (NEP) to the cellular exportin chromosomal maintenance 1 (CRM1). In contrast, the mechanism of vRNP nuclear export, including identification of a protein with NEP functionality, has not been characterized for any thogotoviruses. Here, we characterized vRNP nuclear export in Dhori virus (DHOV), a prototypic member of the *Thogotovirus* genus. DHOV replication and nuclear export of the viral nucleoprotein were inhibited by the canonical CRM1 inhibitor leptomycin B (LMB), suggesting that DHOV vRNP export also utilizes CRM1. Interestingly, LMB treatment led to nuclear retention of the DHOV matrix (M) protein in both infected and transfected cells. Using a mammalian two-hybrid system, we found that DHOV M interacts with CRM1 through a nuclear export sequence (NES) located between amino acids 111 and 128. Mutation of hydrophobic residues within this NES reduced M-CRM1 interaction, abolished the NES phenotype when fused to a fluorescent protein, and impaired rescue of recombinant DHOV by reverse genetics. Together, our results reveal that DHOV vRNP nuclear export is CRM1-dependent and mediated by the M protein rather than a dedicated NEP-like protein, providing the first mechanistic insight into vRNP egress in the genus *Thogotovirus*.

**IMPORTANCE:**

Dhori virus (DHOV) is a pathogenic tick-borne virus in the genus *Thogotovirus*, in the family Orthomyxoviridae. Despite evidence of DHOV exposure in various mammals, including humans, its basic biology is not well understood. We investigated how DHOV’s progeny genome and protein complexes—viral ribonucleoprotein complexes (vRNPs)—are transported out of the nucleus. Our findings show that DHOV, like the influenza viruses, uses the cellular protein chromosomal maintenance protein 1 (CRM1) for vRNP export. We found that chemically inhibiting CRM1 completely blocked DHOV vRNP export, preventing the production of progeny viruses from infected cells. Screening of all known DHOV proteins revealed that the matrix protein, which forms the virus’ scaffold, interacted with CRM1, suggesting it may link CRM1 to the vRNPs. These results advance our understanding of DHOV replication and suggest that chemically inhibiting vRNP export could be a way to treat thogotovirus infections.

## INTRODUCTION

Thogotoviruses are arthropod-borne viruses belonging to the genus *Thogotovirus* within the family *Orthomyxoviridae* (1). This genus is divided into two distinct clades based on sequence homology—the Thogoto thogotovirus (THOV) and the Dhori virus (DHOV) clades (2). Notably, several thogotoviruses—Bourbon virus (BRBV), DHOV, Oz virus (OZV), and THOV—are known to cause severe or fatal illnesses in humans (3–7). Thogotovirus-mediated pathogenicity in humans was first identified in 1966, where the type virus THOV was implicated in two separate cases of human encephalitis in Ibadan, Nigeria, one of which was fatal (3). Additionally, accidental laboratory exposure to DHOV caused similar symptoms with prolonged neurological complications in Russia (4). The close relatives of DHOV—BRBV found in the United States and OZV found in Japan—have also recently been implicated in several human infections, often resulting in death (5–7). Moreover, although documented cases of thogotovirus infections in humans are rare, serological surveys demonstrate high seroprevalence of thogotovirus-neutralizing antibodies in both wild and domestic animals and humans (8–10). However, despite their significant implications for public health and global distribution, the biology of thogotoviruses remains poorly understood.

The genome of thogotoviruses is composed of six segments of single-stranded, negative-sense RNA (1). Segments 1–3 encode for the subunits of the RNA-dependent RNA polymerase (RdRp), consisting of the polymerase basic protein 2 (PB2), polymerase basic protein 1 (PB1), and polymerase acidic protein (PA) subunits. Segment 4 encodes for the glycoprotein (GP), segment 5 encodes for the genome-encapsulating nucleoprotein (NP), and segment 6 encodes for the matrix protein (M) necessary for virion formation and budding. In THOV, segment 6 also encodes for an interferon antagonist (ML) (11–16). Like other orthomyxoviruses, thogotoviruses undergo replication within the nucleus of infected cells. During replication, a copy of negative-sense viral RNA (vRNA) is synthesized, encapsulated by NP, and associated with the trimeric RdRp to form the viral ribonucleoprotein complex (vRNP). To produce infectious progeny, these vRNPs must be first transported out of the nucleus through the nuclear pore complex and into the cytoplasm, where virion assembly and budding of the virion can occur (17).

The process of vRNP nuclear export has been well-characterized for influenza A virus (IAV), the archetypal virus in the family *Orthomyxoviridae*. In IAV, vRNP export relies on the interaction of the nuclear export protein (NEP) produced from splicing of segment 8 mRNA (18, 19), and a ubiquitous cellular exportin—chromosomal region maintenance 1 (CRM1; also known as exportin 1) (18, 20, 21). Two models of NEP/CRM1 interaction have been proposed: the “daisy-chain” model, in which NEP acts as the scaffold linking both CRM1 and the IAV matrix protein (M1) associated with vRNPs, and a variation of this model, wherein NEP additionally interacts with the RdRp (18, 20–26). The interaction between CRM1 and NEP, and any proteinaceous cargo, involves CRM1 recognition of short hydrophobic amino acid motifs known as a nuclear export signal (NES) (18, 23, 27–29). In IAV, NEP contains two redundant NES, both of which are important for vRNP nuclear export, while NP additionally has three NESs, one of which is solely CRM1-dependent (18, 23, 30). Based on the similarity of their life cycle to influenza viruses, thogotoviruses’ vRNP nuclear export can be speculated to be mediated by a viral protein. However, a protein with NEP functionality has yet to be identified in any member of the *Thogotovirus* genus, and the mechanism of vRNP nuclear export in thogotoviruses’ life cycle remains elusive.

In this study, we investigated the mechanism of vRNP nuclear export in DHOV. Our findings demonstrated that CRM1 inhibition by the canonical CRM1 inhibitor leptomycin B (LMB) inhibited DHOV replication via retention of vRNPs within the nucleus of infected cells. All DHOV proteins were evaluated for their role in this mechanism, revealing that DHOV M interacts with CRM1 through an NES. Importantly, mutating key residues within this NES reduced DHOV M binding to CRM1 in a mammalian two-hybrid system. Rescued recombinant DHOV (rDHOV) carrying mutations in the DHOV M NES were also either nonviable or attenuated relative to wild-type (WT) virus. Altogether, these results suggest that DHOV vRNP export is accomplished via interaction between DHOV M and CRM1, representing the first characterization of vRNP nuclear export in a member of the genus *Thogotovirus*.

## RESULTS

### CRM1 inhibition diminishes DHOV replication

**Fig 1:**
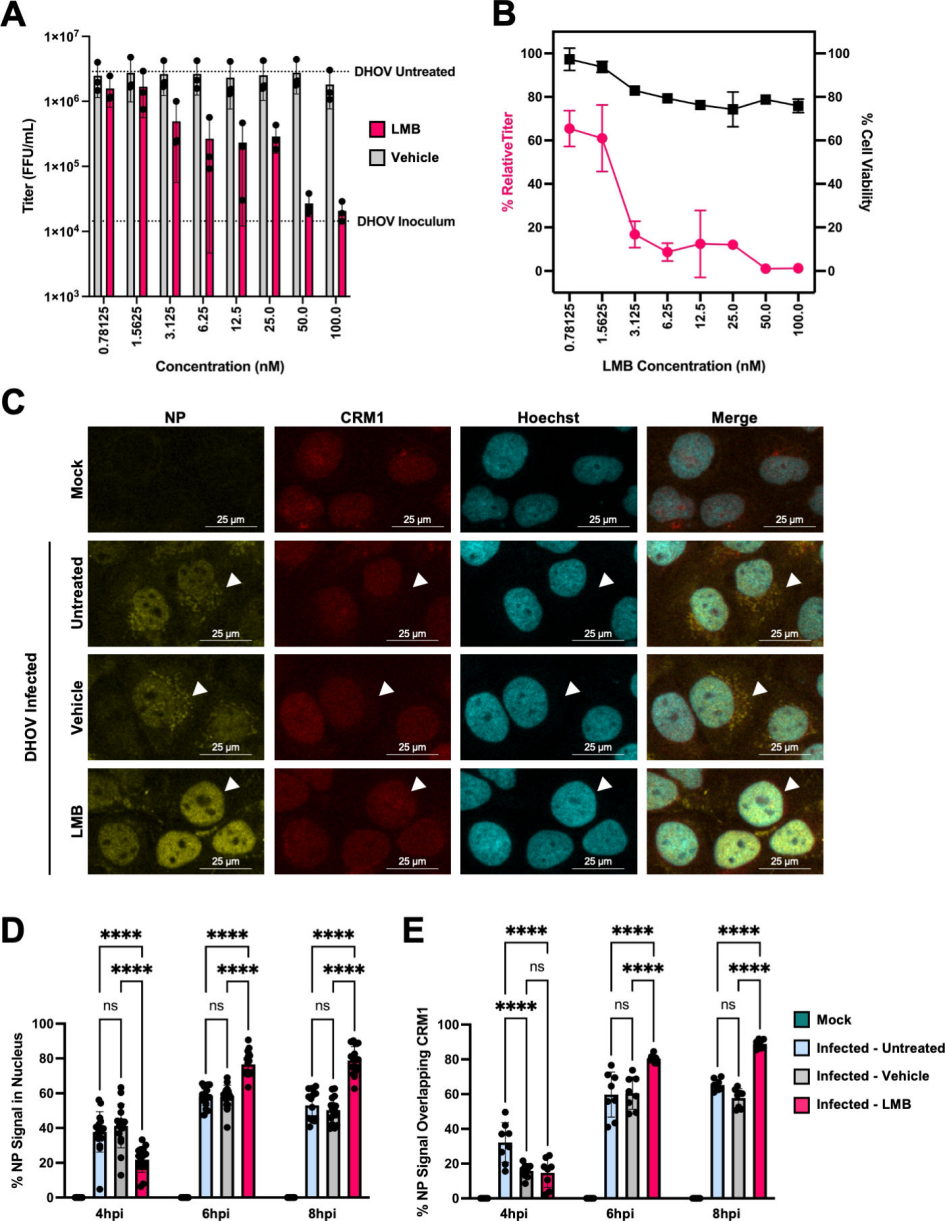
Leptomycin B (LMB) causes a dose-dependent reduction in Dhori virus (DHOV) titer and viral ribonucleoprotein (vRNP) retention in the nucleus. (**A**) DHOV titer in Huh7 cells (MOI = 0.1) with treatment with the indicated dose of LMB or vehicle control. Virus infectivity titers in supernatants are shown in focus-forming unit (FFU)/mL. Dashed lines indicate the residual DHOV titer following infection and washing with SFM (DHOV inoculum) and DHOV titer from untreated cells (DHOV untreated). (**B**) (left, magenta Y-axis) DHOV titer from LMB-treated cells expressed as a percentage of vehicle control-treated cells. (right, black Y-axis) Cell viability of Huh7 cells treated with the indicated dose of LMB expressed as a percentage of the corresponding dose of vehicle control. (**C**) Localization of DHOV NP and CRM1 in Huh7 cells at 6 hours post-infection (hpi) (MOI = 100) treated with 100 nM of LMB, vehicle control, or untreated, examined by immunofluorescence using anti-CRM1 and custom anti-DHOV NP antibodies. Arrowheads highlight contrast in NP signal localization. Nuclei stained using Hoechst 33342. Images taken using a confocal microscope (ZEISS LSM 980 with Airyscan2) using 10× objective. (**D**) Quantification of the percentage of the NP signal overlapping the nuclei using the Just Another Colocalization (JaCoP) plugin in ImageJ. (**E**) Quantification of the percentage of NP signals overlapping the CRM1 signal using the JaCoP plugin in ImageJ. ns > 0.05, ****P ≤ 0.0001; ordinary one-way ANOVA.

First, the effect of inhibiting CRM1 with LMB, a canonical CRM1 inhibitor (31–33), on DHOV replication was investigated in the permissive cell line human hepatoma Huh7 (34). LMB treatment caused a dose-dependent reduction in DHOV titer, achieving approximately a 2-log decrease (over 90% reduction) compared to the vehicle control at a dose of 50 nM (Fig. 1A and B). Importantly, the DHOV titer at a dose of 50 nM was equivalent to the titer from culture supernatant samples harvested immediately after 1 hour viral adsorption, suggesting near-complete inhibition of the release of progeny viruses. Significant reductions in DHOV titer were observed at even lower LMB doses, with a calculated IC_50_ of 2.96 nM. LMB-treated cells maintained a cell viability of 70% or greater relative to the vehicle control across all tested doses (Fig. 1B). These results indicate that LMB inhibits DHOV replication, suggesting that CRM1 plays a critical role in the DHOV life cycle.

### LMB treatment causes DHOV vRNPs to be retained in the nucleus

Next, we examined whether DHOV vRNPs are retained in the nucleus upon CRM1 inhibition by LMB, as is the case in IAV (33). In untreated and vehicle-treated cells, perinuclear dot-like signals of DHOV NP were detected outside the nucleus starting at 6 hours post-infection (hpi), indicating that vRNP export had initiated by this time point (Fig. 1C, arrowheads; see Fig. S1A and B at https://doi.org/10.5281/zenodo.17715494). In contrast, perinuclear localization of NP was not observed in LMB-treated cells at either 6 or 8 hpi (Fig. 1C; see Fig. S1B at https://doi.org/10.5281/zenodo.17715494). Analysis of these images revealed a statistically significant increase in the colocalization of NP with both the nucleus and CRM1 in LMB-treated cells at 6 and 8 hpi, compared to vehicle and untreated controls (Fig. 1D and E). These results strongly suggest that LMB inhibition of CRM1 induces DHOV vRNP retention in the nucleus, thereby inhibiting viral replication.

### The cellular distribution of DHOV M changes with LMB treatment, resulting in its nuclear retention

**Fig 2:**
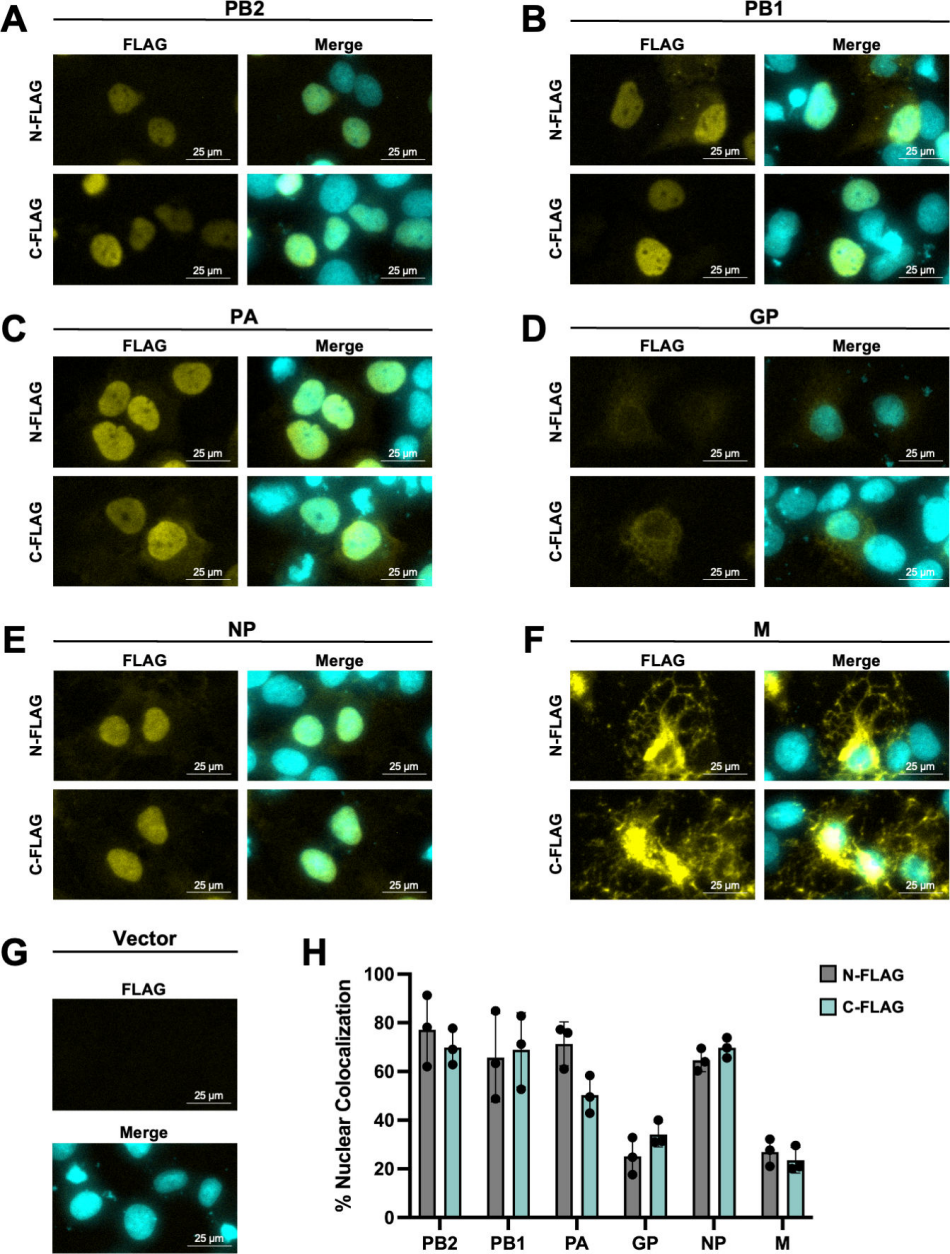
Localization of FLAG-tagged DHOV proteins in a single-protein transfection system. (**A–G**) Huh7 cells were transfected with 1.0 µg of the indicated pCAGGS expression construct expressing DHOV (**A**) PB2, (**B**) PB1, (**C**) PA, (**D**) GP, (**E**) NP, (**F**) M, or (**G**) empty vector. After 24 hours transfection, cells were fixed, and immunofluorescence was performed against the FLAG epitope. Nuclei were stained using Hoechst 33342. Images taken using a ZOE Fluorescent Cell Imager using a 20× objective. (**H**) Quantification of the overlap of FLAG tag signal with the nuclei from conditions A–F using the JaCoP plugin in ImageJ.

**Fig 3:**
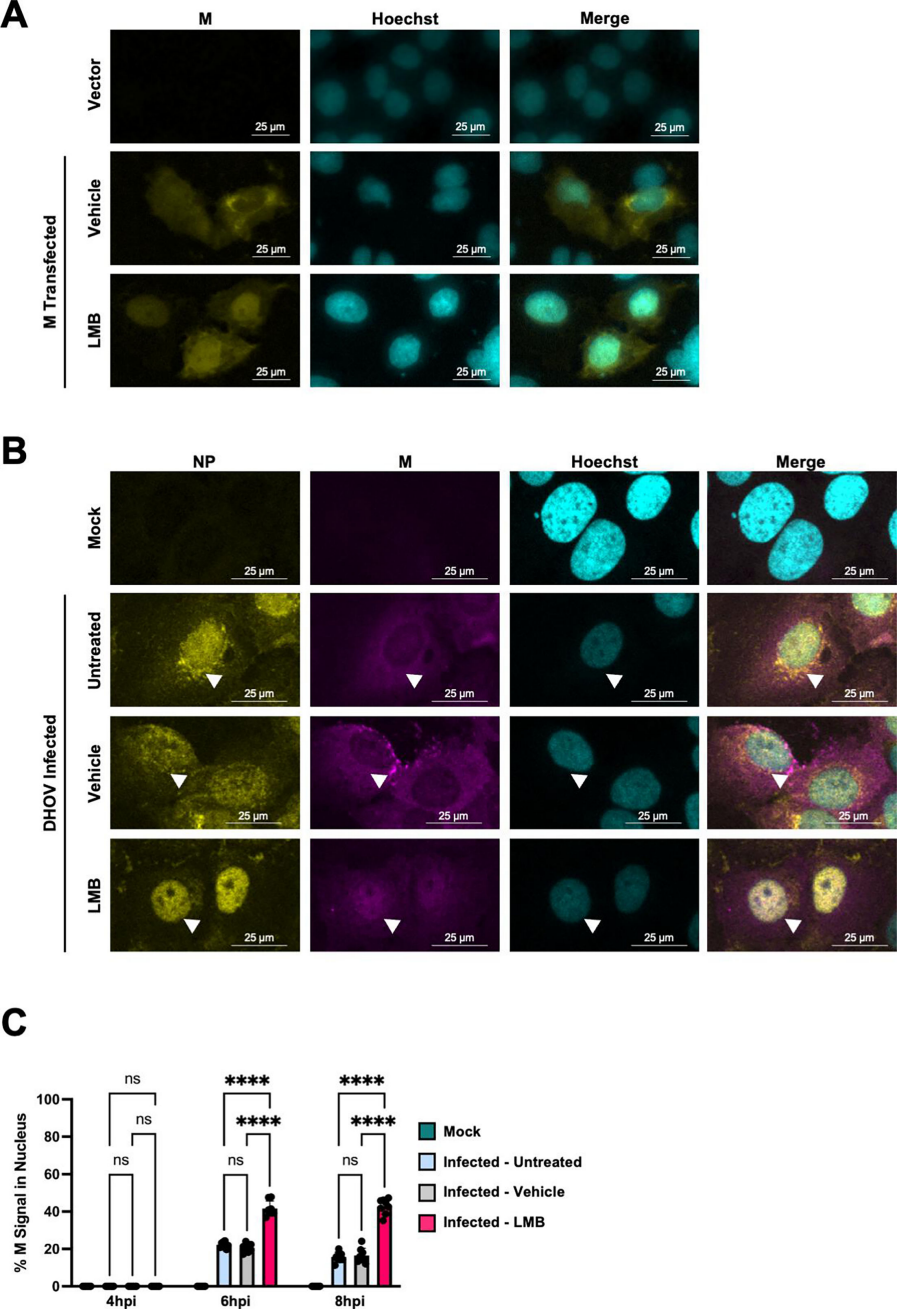
DHOV M alters localization upon LMB treatment. (**A**) Huh7 cells were transfected with 1.0 µg of the pCAGGS vector expressing N-terminally FLAG-tagged DHOV M. After 24 hours transfection, cells were treated with 100 nM LMB, vehicle control, or untreated media for 1 hour and then fixed. Immunofluorescence conducted using custom anti-BRBV M antibody. Images taken using a ZOE Fluorescent Cell Imager using a 20× objective. (**B**) Localization of DHOV NP and M in Huh7 cells at 6 hpi (MOI = 100) treated with 100 nM of LMB, vehicle, or untreated media examined by immunofluorescence using custom anti-DHOV NP and anti-BRBV M antibodies. Arrowheads highlight contrast in NP signal localization. Images taken using a confocal microscope (ZEISS LSM 980 with Airyscan2) using 10× objective. (**C**) Quantification of the percentage of M signals overlapping the nuclei in infected cells using the JaCoP plugin in ImageJ. Nuclei stained using Hoechst 33342. ns > 0.05, ****P ≤ 0.0001; ordinary one-way ANOVA.

Previous studies have shown that singly expressed IAV NEP can be detected in both the nucleus and cytoplasm due to its ability to shuttle between these two compartments (35). To identify DHOV protein(s) with this same shuttling potential, six DHOV proteins (PB2, PB1, PA, GP, NP, and M) were individually expressed with a FLAG tag placed at either the N- or C-terminus of each ORF, and their cellular distribution was assessed. Regardless of FLAG tag placement, NP and the RdRp subunits, PB2, PB1, and PA, were predominantly nuclear-localized, while GP was predominantly cytoplasmic (Fig. 2A through E and G,H). In contrast, M displayed a diffuse localization pattern throughout the nucleus and cytoplasm (Fig. 2F though H), suggesting that DHOV M may be capable of shuttling between these compartments. Notably, upon LMB treatment, M became retained in the nucleus along with CRM1, in contrast with the diffuse localization observed in cells treated with the vehicle control (Fig. 3A). These findings suggest that CRM1 is necessary for M to shuttle from the nucleus to the cytoplasm.

The cellular distribution of DHOV M, with or without CRM1 inhibition by LMB, was further examined in DHOV-infected cells. In untreated and vehicle-treated cells, DHOV M localization mirrored the perinuclear signal of the DHOV NP during vRNP export beginning at 6 hpi (Fig. 3B, arrowheads; see Fig. S2A and B at https://doi.org/10.5281/zenodo.17715494). In contrast, LMB treatment caused noticeable retention of both M and NP within the nucleus at both 6 and 8 hpi (Fig. 3B, arrowheads; see Fig. S2B at https://doi.org/10.5281/zenodo.17715494). Analysis of these images showed a statistically significant increase in the colocalization of M with the nucleus in LMB-treated cells, compared to both vehicle and untreated cells beginning at 6 hpi (Fig. 3C). These results strongly suggest that DHOV M is involved in the nuclear export of vRNPs.

### DHOV M interacts with CRM1 in a mammalian two-hybrid system

**Fig 4:**
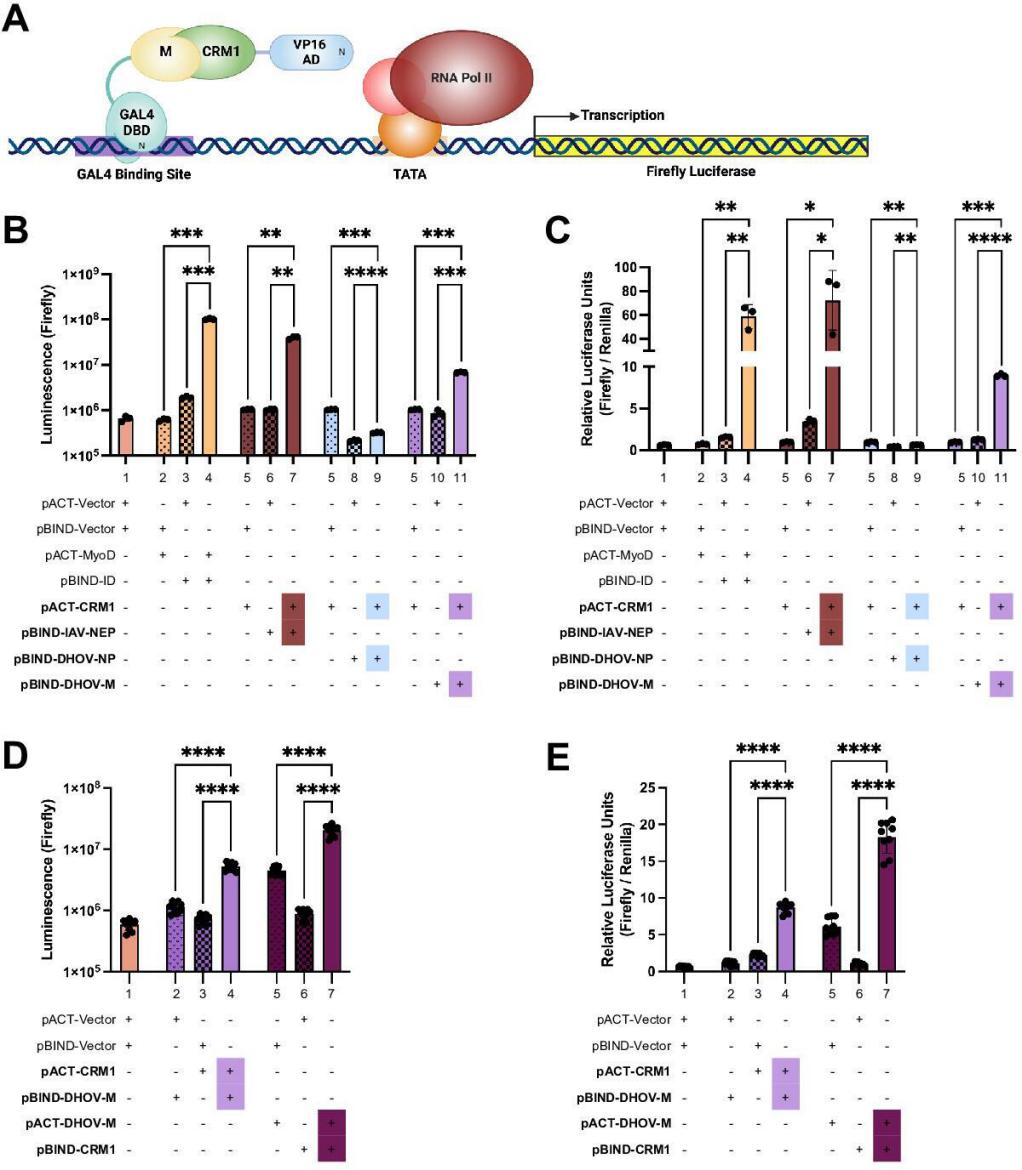
DHOV M but not NP interacts with CRM1 in a mammalian two-hybrid system. (**A**) Schematic depicting the Promega CheckMate mammalian two-hybrid system using the pBIND (VP16) CRM1 fusion orientation. (**B–E**) CheckMate system luciferase data. HEK-293 cells were co-transfected with 100 ng of the pG5*luc* reporter plasmid and combinations of the indicated pACT and pBIND plasmids. After 96 hours post-transfection, cell lysates were harvested, and luciferase assay was performed. *Renilla* luciferase, expressed from the pBIND vector under a constitutive promoter, was used to normalize firefly luciferase data for transfection efficiency. Interaction between pACT-MyoD and pBIND-ID (Promega) was used as a positive control. (**B–C**) Human CRM1 fused to the VP16 transcriptional activation domain (pBIND) and indicated viral protein fused to the GAL4 DNA-binding domain (pACT). (**D–E**) Orientation comparison demonstrating comparing the luciferase activity of pBIND-CRM1 and pACT-DHOV-M and vice versa. (**B and D**) Raw firefly luciferase data. (**C and E**) Firefly luciferase data normalized to transfection efficiency (Firefly/*Renilla*). Results shown represent at least three independent experiments. Lane 5 repeated in B and C for easier interpretation. ns > 0.05, *P ≤ 0.05, **P ≤ 0.01, ***P ≤ 0.001, ****P ≤ 0.0001; *t*-test. (**A**) Created in BioRender. Swenson, V. (2023) https://BioRender.com/b16s227.

Given that the interaction between CRM1 and IAV NEP is critical for IAV vRNP export (21, 36), the interaction between DHOV M and CRM1 was assessed using the Promega CheckMate System—a luciferase reporter-based mammalian two-hybrid system that quantitatively evaluates protein:protein interaction in mammalian cells via fusion to either the GAL4 DNA-binding domain or VP16 transcriptional activation domain (Fig. 4A) (37). Our results indicated that, similar to the positive control MyoD and ID (Fig. 4B and C, lanes 2–4), co-transfection of CRM1 and IAV NEP induced significant luciferase reporter activity compared to corresponding vector controls (Fig. 4B and C, lanes 5–7), consistent with previous findings (23). Co-transfection of DHOV M and CRM1 induced a statistically significant increase in firefly luciferase expression over corresponding vector controls, suggesting that these two proteins interact *in vitro* (Fig. 4B and C, lanes 5, 10–11). Importantly, this interaction was seen regardless of fusion protein orientation, with the opposite orientation (pACT/GAL4 DHOV M) also inducing a statistically significant increase in firefly luciferase expression over vector controls (Fig. 4D and E, lanes 5–7), suggesting that DHOV M interacts with CRM1. Due to the presence of a CRM1-dependent NES within IAV NP (36), DHOV NP was also tested for its potential interaction with CRM1. However, co-transfection of DHOV NP and CRM1 did not cause a statistically significant increase in firefly luciferase expression over both vector controls (Fig. 4B and C, lanes 5, 8–9), suggesting a lack of interaction between these two proteins.

### Serial truncations of DHOV M identify a region with nuclear export capability

**Fig 5:**
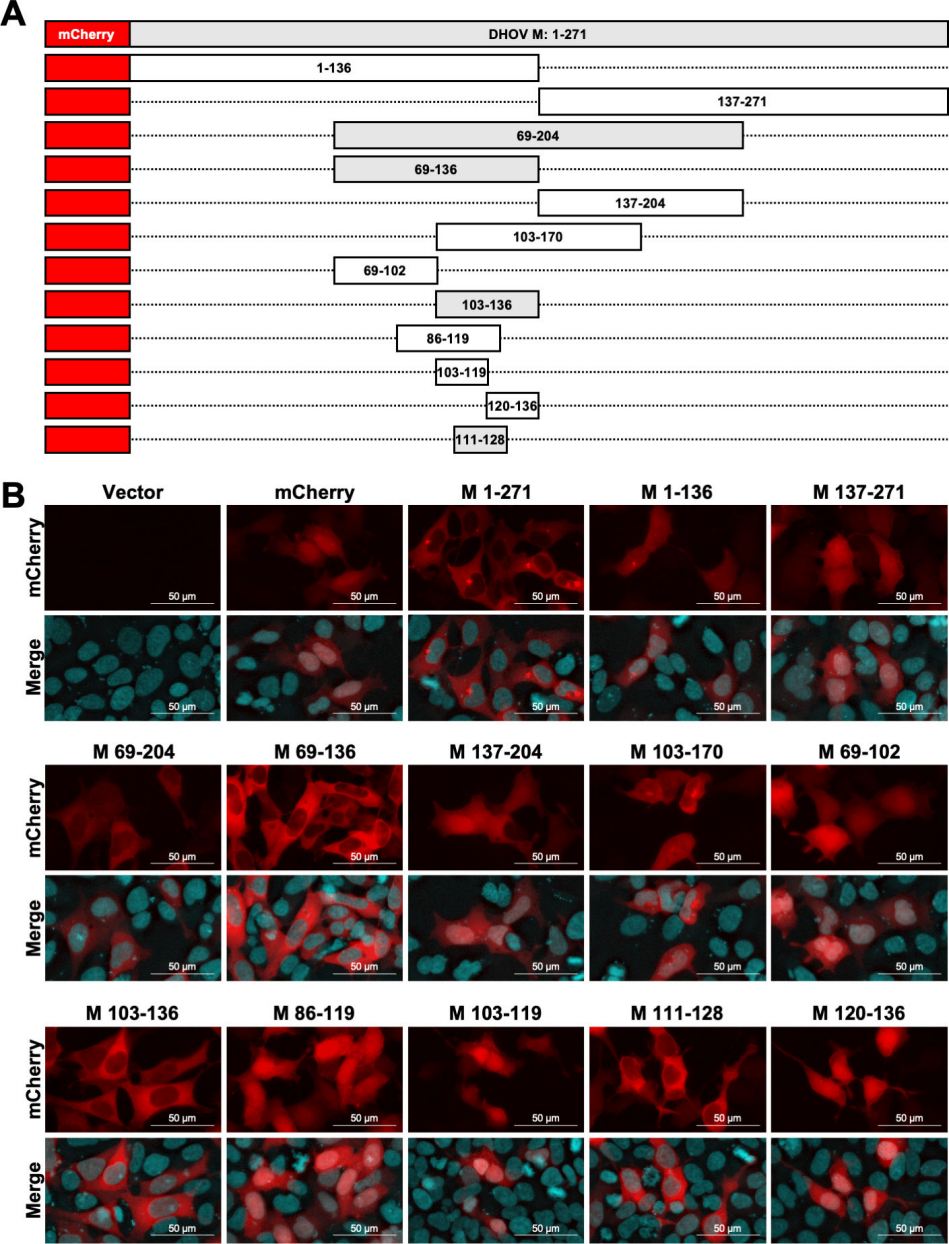
DHOV M contains a putative NES that can cause nuclear export of mCherry. (**A**) Schematic of successive truncations to DHOV M ORF fused to the C-terminus of mCherry. Numbers indicate amino acid residues. (**B**) Immunofluorescence images showing localization of the indicated mCherry–DHOV M fusion construct. HEK-293 cells were transfected with 0.5 µg of the pCAGGS vector expressing indicated fusion construct. After 24 hours transfection, cells were fixed with 10% neutral buffered formalin, and protein localization was assessed. Nuclei were stained using Hoechst 33342. Images were taken using a Nikon Eclipse Ts2 microscope with an Excelis MPX-6 camera under 20 × magnification.

The nuclear export capability of DHOV M was further examined by fusing M with the fluorescent protein mCherry (Fig. 5A) (36, 38, 39). While mCherry alone predominantly localized to the nucleus, the mCherry signal clearly shifted to the cytoplasm when fused to full-length M (Fig. 5B), indicating that DHOV M can mediate the export of this fusion protein from the nucleus to the cytoplasm. To identify the region within M responsible for nuclear export, we constructed a series of truncated M proteins fused to mCherry (Fig. 5A) and assessed their localization within transfected HEK-293 cells. The M ORF was initially divided into three fragments, spanning amino acids 1–136, 69–204, and 137–271 (Fig. 5A). When these truncation fusion proteins were visualized, only fragments 69–204 localized to the cytoplasm, whereas 137–271 remained in the nucleus. In contrast, fragments 1–136 displayed a diffuse distribution across both the nucleus and cytoplasm (Fig. 5B). Subdivision of amino acids 69–204 identified that fragments 69–136 predominantly localized to the cytoplasm, while fragments 103–170 and 137–204 showed minimal cytoplasmic localization. Further subdivision of amino acids 69–136 revealed that only 103–136 exhibited a clear cytoplasmic localization, like the pattern observed with full-length M, while 69–102 and 86–119 remained in the nucleus. Finally, division of 103–136 demonstrated that the 18 amino acid stretch spanning residues 111–128 retained the WT M localization pattern, whereas fragments 103–119 and 120–136 showed a distribution like WT mCherry (Fig. 5A and B). These results suggest that amino acids 111–128 within the DHOV M ORF contain a signal required for mediating nuclear export.

### DHOV M contains a putative LMB sensitive NES within amino acids 111–128

**Fig 6:**
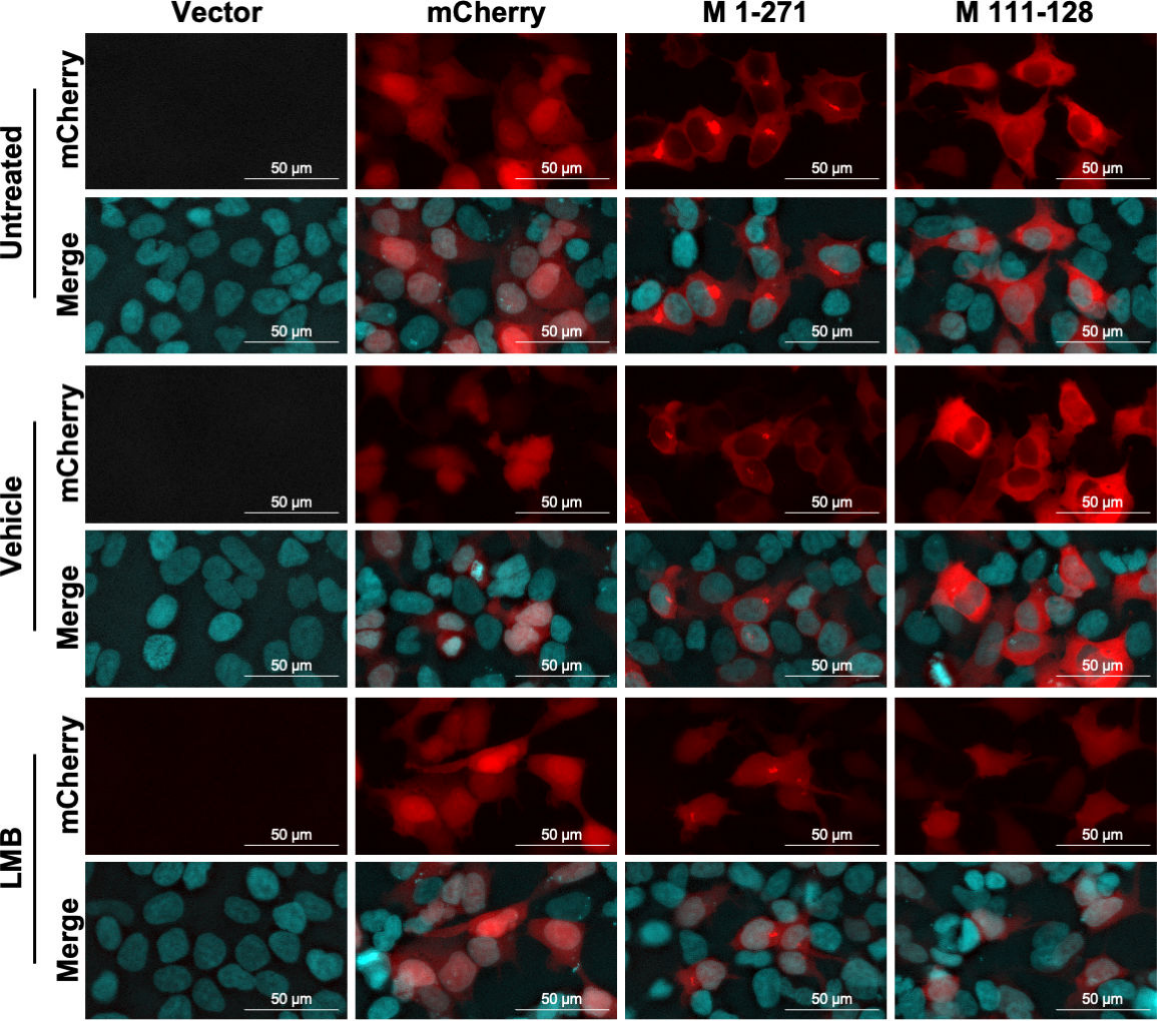
Both full-length DHOV M and M 111–128 are retained in the nucleus upon LMB treatment. Immunofluorescence images showing localization of the indicated mCherry – DHOV M fusion construct. HEK-293 cells were transfected with 0.5 µg of the pCAGGS vector expressing indicated fusion construct. After 24 hours transfection, cells were treated for 2 hours with media containing 100 µg/µL cycloheximide and 100 nM LMB, vehicle control, or untreated media and then fixed. Nuclei stained using Hoechst 33342. Images taken using a Nikon Eclipse Ts2 microscope with an Excelis MPX-6 camera under 20 × magnification.

Given that full-length DHOV M was sensitive to LMB treatment in both transfected and infected cells (Fig. 3A through C; see Fig. S2B at https://doi.org/10.5281/zenodo.17715494), we next investigated whether residues 111–128 alone were similarly sensitive to CRM1 inhibition. As previously demonstrated, both full-length M and the 111–128 fragment fused to mCherry localized to the cytoplasm of transfected cells in untreated and vehicle-treated controls (Fig. 6). However, treatment with LMB led to nuclear retention of both WT M and the 111–128 fragment, indicating their dependence on CRM1-mediated nuclear export (Fig. 6). These results suggest that amino acids 111–128 within the DHOV M ORF contain a CRM1-dependent NES.

**Fig 7:**
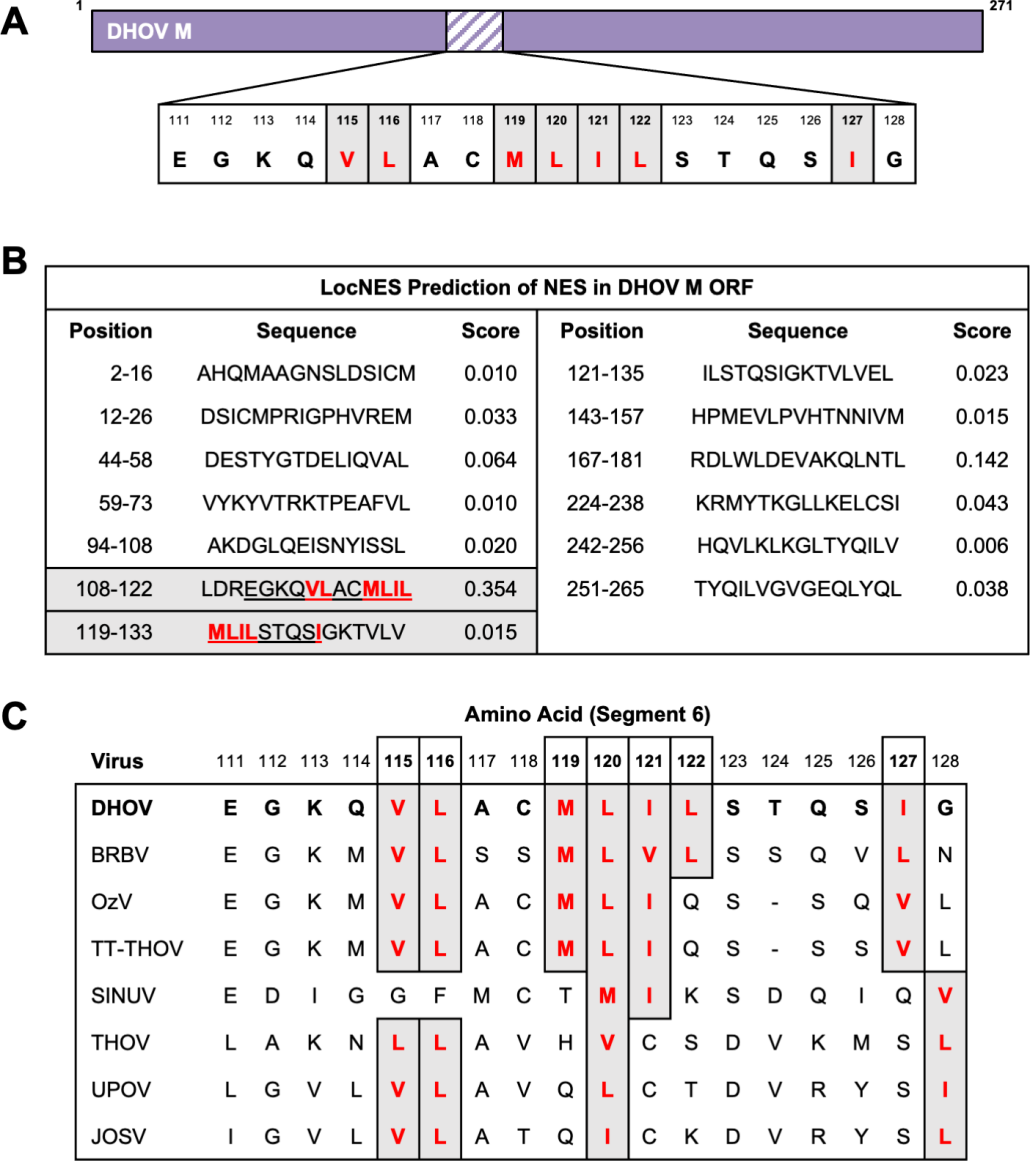
Identification and conservation of NES1 among thogotoviruses. (**A**) Schematic showing the NES1-containing region within DHOV M ORF. Hydrophobic amino acids within NES1 highlighted. (**B**) Predicted NES within the DHOV M ORF, as identified by LocNES (40). Hydrophobic amino acids within NES1 are highlighted. (**C**) Conservation of NES1 hydrophobic amino acids among segment 6 ORF of various thogotoviruses. Alignment created using the amino acid sequence of segment 6 of the indicated thogotoviruses using CLUSTAL W.

The nuclear export of proteinaceous cargo through CRM1 is facilitated by the interaction between the NES within the cargo and the NES-binding groove of CRM1 (27). NES sequences can be identified by specific patterns of hydrophobic residues, generally following the consensus sequence Φ1-(X)_2-3_-Φ2-(X)_2-3_-Φ3-X-Φ4, where “X” denotes any amino acid and “Φ” denotes any hydrophobic amino acid (Leu, Ile, Val, Met, or Phe) (27). Although seven hydrophobic amino acids are present within DHOV M 111–128 (Fig. 7A), their distribution and spacing made it challenging to identify which residues could be essential for NES function. However, this region was highlighted as the highest-scoring NES candidate by the prediction tool LocNES (Fig. 7B) and was found to be highly conserved among thogotoviruses, particularly within the DHOV clade (Fig. 7C). Collectively, these findings suggest that the hydrophobic residues within DHOV M 111–128 (hereafter referred to as NES1) may form a highly evolutionarily conserved NES (40).

### Mutation of the hydrophobic amino acids within DHOV M NES1 disrupts interaction with CRM1 and reverses NES phenotype

**Fig 8:**
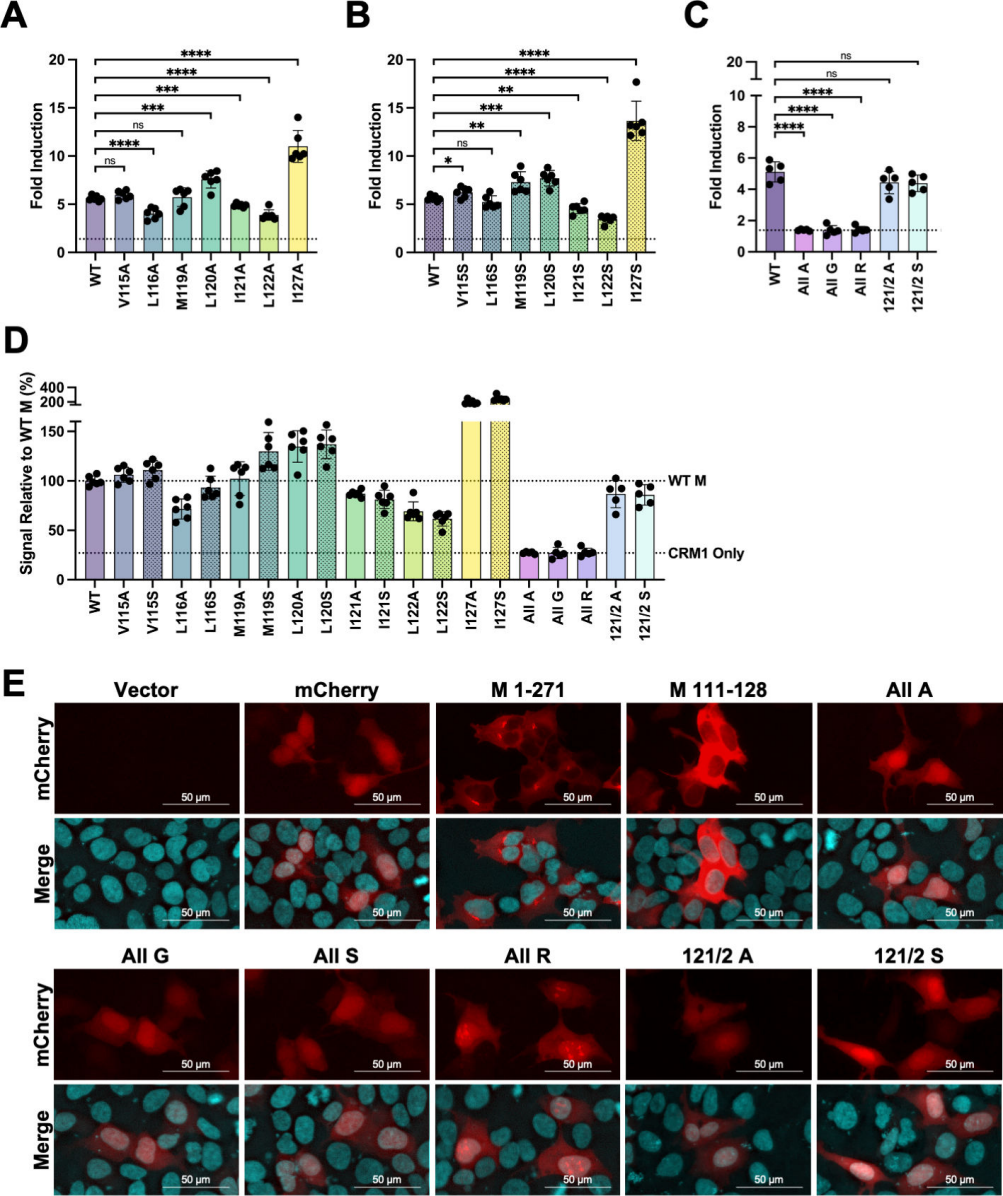
Mutation of NES1 residues within DHOV M reduces interaction with CRM1 in a mammalian two-hybrid system and abrogates the NES phenotype. (**A–D**) CheckMate system luciferase data. HEK-293 cells were co-transfected with 100 ng of the pG5*luc* reporter plasmid and combinations of other plasmids as indicated. After 96 hours transfection, cell lysates were harvested, and luciferase assay was performed. Interaction between pACT-CRM1 and pBIND-DHOV-M was used as a positive control. (**A–C**) Data shown as firefly luciferase data normalized to transfection efficiency (Firefly/*Renilla*) and then to vector control (sample/vector). The dashed line indicates the system background of pACT CRM1 alone. (**A**) Alanine point mutants. (**B**) Serine point mutants. (**C**) Combinatorial mutants. (**D**) A–C expressed as percentage of WT DHOV-M signals. Dashed lines indicate the signal of WT M (WT M) and pACT CRM1 alone (CRM1 Only). Results shown represent at least five independent experiments. (**E**) Immunofluorescence images showing localization of the indicated mCherry–DHOV M fusion construct. All mutants shown (All A, All G, All S, All R, 121/2A, and 121/2S) contain the specified mutations within DHOV M 111–128 fused to mCherry. HEK-293 cells were transfected with 0.5 µg of the pCAGGS vector expressing the indicated fusion construct and fixed 24 hours post-transfection. Nuclei were stained using Hoechst 33342. Images taken using a Nikon Eclipse Ts2 microscope with an Excelis MPX-6 camera under 20 × magnification. ns > 0.05, *P ≤ 0.05, **P ≤ 0.01, ***P ≤ 0.001, ****P ≤ 0.0001; (**A–C**) *t*-test.

To investigate the role of DHOV M NES1 in mediating interaction with CRM1, each of the seven hydrophobic residues, V115, L116, M119, L120, I121, L122, and I127, were individually mutated to either another hydrophobic amino acid (alanine) or a hydrophilic amino acid (serine). The resulting DHOV M mutants were then analyzed using the Promega CheckMate System to assess their ability to induce firefly luciferase activity, suggesting CRM1 binding. Co-transfection of all NES1 mutants with CRM1 resulted in an increase in firefly luciferase expression over vector controls (Fig. 8A and B; see Fig. S3 at https://doi.org/10.5281/zenodo.17715494). Among all mutants, only substitutions at I121 and L122 consistently resulted in a statistically significant decrease in firefly luciferase expression, regardless of whether alanine or serine was used (Fig. 8A and B; see Fig. S3 at https://doi.org/10.5281/zenodo.17715494). When these data were normalized to WT M, the signal from the I121 mutants was reduced by more than 10% relative to WT levels, while the L122 mutants showed an even greater reduction, with signals below 70% of those seen in WT M (Fig. 8D).

To further investigate the contribution of the hydrophobic residues with DHOV M NES1 to surrogate CRM1 interaction within the Promega CheckMate System, we generated double mutants targeting the residues whose mutations caused the greatest reduction in firefly luciferase activity (121/2A and 121/2S). Additionally, to evaluate the effect of simultaneously mutating all hydrophobic residues within NES1, we generated pan mutants in which all seven hydrophobic residues were replaced with either alanine (All A), glycine (All G), or arginine (All R), representing a small side chain, no side chain, or hydrophilic replacements, respectively. Interestingly, although the double mutants 121/2A and 121/2S displayed slightly reduced firefly luciferase activity relative to WT M, the reduction did not exceed that achieved by individual point mutations of I121 or L122 (Fig. 8A through D; see Fig. S3 at https://doi.org/10.5281/zenodo.17715494). However, pan-mutation of all hydrophobic residues completely abolished firefly luciferase induction, reducing the activity to background levels regardless of the substituted amino acid (Fig. 8C and D; see Fig. S3 at https://doi.org/10.5281/zenodo.17715494). Together, these results indicate that the hydrophobic residues within DHOV M NES1—particularly I121 and L122—may be key contributors to CRM1 interaction.

Building on our earlier observation that fusing DHOV M NES1 to mCherry reproduces an NES phenotype, we next evaluated how mutations within NES1 impact the subcellular distribution of mCherry-DHOV M NES1 fusion proteins. Double mutation of residues I121 and L122, as well as pan mutation of all hydrophobic residues to alanine, glycine, serine (All S), or arginine, was tested in our mCherry fusion protein system. Remarkably, both the double and pan mutants resulted in a complete reversal of the NES phenotype, resulting in fusion proteins that displayed a diffuse distribution across the nucleus and cytoplasm, like that of WT mCherry (Fig. 8E). Collectively, these results suggest a critical role of NES1 in mediating DHOV M interaction with CRM1 and NES functionality.

### Mutations in NES1 attenuate or abolish the rescue of DHOV from a reverse genetics system

**Fig 9:**
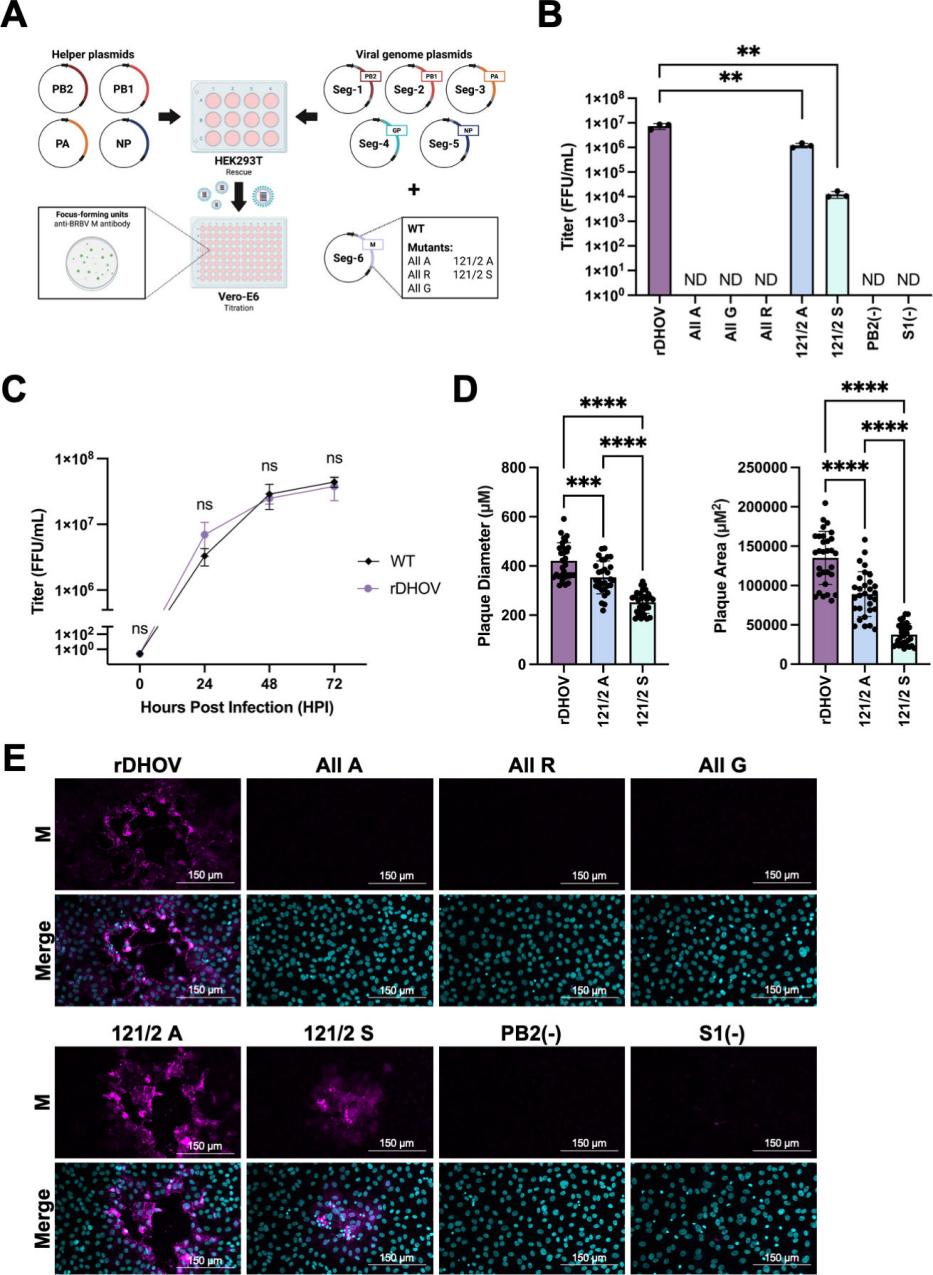
Mutations of NES1 residues attenuate rDHOV. (**A**) Schematic of the strategy to rescue rDHOV. HEK-293T cells were co-transfected with 50 ng of indicated helper plasmids (pCAGGS) and 125 ng of indicated viral genome plasmids (pPolI). The supernatant was placed onto Vero-E6 cells for titration using the custom anti-BRBV M antibody. (**B**) Titer of rescued rDHOV expressed as focus-forming unit (FFU)/mL. ND = not detected (**C**) Growth curve of WT DHOV and rDHOV. Titer expressed as FFU/mL. (**D**) Plaque diameter and area of indicated rDHOV measured using ImageJ, with 30 plaques quantified per condition. (**E**) Images of representative rDHOV and rDHOV mutant plaques on Vero-E6 cells. Immunofluorescence assay was performed using custom anti-BRBV M antibody. Nuclei stained using Hoechst 33342. Images taken using a confocal microscope (ZEISS LSM 980 with Airyscan2) using 10× objective. Results shown represent three independent experiments. ns > 0.05, **P ≤ 0.01, ***P ≤ 0.001, ****P ≤ 0.0001; (**B and C**) *t*-test; (**D**) one-way ANOVA. (**A**) Created in BioRender. Swenson, V. (2025) https://BioRender.com/ecm2b8d.

Finally, the importance of the hydrophobic residues within NES1 was evaluated within the context of the viral life cycle using infectious DHOV. A human RNA polymerase I-driven reverse genetics system for DHOV was developed (Fig. 9A) and employed to rescue recombinant DHOV (rDHOV) as well as rDHOV mutants carrying either double (121/2A, 121/2S) or pan (All A, All G, All R) mutations within the hydrophobic residues of DHOV M NES1. Infectious rDHOV was successfully rescued only when all 10 plasmids were co-transfected in HEK-293T cells, but not when segment 1 (S1) or PB2 was absent (Fig. 9B). When the growth kinetics of WT DHOV was compared to rDHOV, no statistically significant differences in viral growth were observed at any time point (Fig. 9C), validating the biological functionality of rDHOV.

Although the double mutants 121/2A and 121/2S were successfully rescued, their rescue titers were consistently lower than that of rDHOV, with 121/2A yielding a half-log, and 121/2 S a nearly three-log decrease in rescue efficiency (Fig. 9B). In contrast, rDHOV carrying pan mutations could not be rescued, irrespective of the amino acid substitutions introduced (Fig. 9B). To further quantify the attenuation of 121/2A and 121/2S, we measured both the plaque diameter and area of rDHOV on Vero-E6 cells. Both the plaque diameter and area of rDHOV 121/2A and 121/2S were significantly decreased compared to rDHOV, with 121/2S producing plaques only one-fourth the size of those formed by rDHOV (Fig. 9D and E). Collectively, these results suggest that mutating hydrophobic residues within DHOV M NES1—particularly I121 and L122—attenuates the resulting rDHOV, supporting a role for DHOV M NES1 in the viral life cycle through with CRM1.

## DISCUSSION

In this study, we present a CRM1-dependent mechanism for the nuclear export of DHOV vRNPs, mediated by the M protein. Since the identification of NEP in IAV (18, 21), viral proteins with NEP functionality have been recognized across various orthomyxoviruses, including members of *Betainfluenzavirus, Gammainfluenzavirus,* and *Deltainfluenzavirus* genera (IBV, ICV, and IDV), as well as the *Isavirus*, *Mykissvirus,* and possibly *Sardinovirus* genera (41–44). Our study is the first to characterize the molecular mechanism of vRNP export in the genus *Thogotovirus,* identifying DHOV M as an analog of IAV NEP. This finding proposes a novel concept that DHOV M has dual functionality, mediating both viral budding (45) and vRNP nuclear export within a single protein. This condensation of protein functionality could explain how members of the *Thogotovirus* genus compensate for the loss of two segments of coding capacity relative to IAV/IBV.

The nuclear export of newly formed vRNPs is a crucial step in the viral life cycle of the family *Orthomyxoviridae* (17). During the formation of IAV vRNP export complexes, NEP interacts with CRM1 through its N-terminal NES, while the C-terminus of NEP binds to M1 bound to the vRNP (18, 21, 23–26, 46, 47). The interaction between NEP and M1 is thought to require NEP interaction with the RdRp, which strengthens the binding of M1 to the vRNP (25, 47). Interestingly, no direct interaction between IAV M1 and CRM1 has been reported (20); however, our findings indicate that DHOV M interacts with CRM1 (Fig. 4). Thus, DHOV M may combine the functionality of both NEP and M1 of IAV by bridging the gap between the vRNP complex and CRM1. Notably, we observed colocalization between M and NP during vRNP export in infected cells and retention of both DHOV NP and M within the nucleus upon LMB treatment (Fig. 3), suggesting that M plays a critical role in vRNP export through its interaction with CRM1.

Successive truncations of the DHOV M ORF fused to mCherry identified a region spanning amino acids 111–128 that contains a cluster of seven hydrophobic residues and was sensitive to LMB treatment (Fig. 5 to 7). While most individual alanine and serine substitutions at each of these residues impaired the interaction between M and CRM1 in a mammalian two-hybrid system, mutations at residues 121 and 122 had the most pronounced negative impact (Fig. 8A through D). The negative impact of the NES1 mutation was further confirmed by pan mutation of all seven hydrophobic residues to alanine, glycine, or arginine, all of which abolished interaction with CRM1 in a mammalian two-hybrid system and reversed the NES phenotype in fusion to mCherry (Fig. 8C through E). rDHOV carrying pan mutations of hydrophobic residues in NES1 could not be rescued using our reverse genetics system (Fig. 9B), whereas rDHOV 121/2A and 121/2S mutants were successfully rescued, but exhibited attenuation relative to WT rDHOV, with 121/2S showing the greatest level of attenuation.

The successful rescue of rDHOV 121/2A and 121/2S may be attributed to the presence of nearby hydrophobic residues, such as M119 and L120, which could compensate for the mutations of I121 and L122. Furthermore, the similarity between the side chains of alanine and the original methionine and leucine at residues 119 and 120 may enable DHOV M 121/2A to retain greater functionality, whereas substitution with the hydrophobic serine in 121/2S diminishes this activity. The sensitivity of amino acid selection in NES mutagenesis was demonstrated by Huang et al., who showed that IAV NEP NES2 point mutations to hydrophobic residues (F35A or L38A) still allowed mutant IAV to be rescued, whereas substitution with a hydrophilic amino acid (F35K or L38R) abolished the rescue (23). Further studies are required to identify additional potential NES within DHOV M and elucidate the full structure of the DHOV vRNP export complex.

Interestingly, although we demonstrated the presence of an NES between amino acids 111 and 128 within the M ORF, the M fragments 1–136 and 103–170 fused to mCherry showed some nuclear localization (Fig. 5B). One possible explanation for this discrepancy is the presence of an NLS within the N-terminus of DHOV M, as has been reported for IAV M1 (48). Screening of the DHOV M ORF for canonical NLS did not identify any obvious NLS sequence (data not shown). However, the nuclear localization of DHOV M upon LMB treatment (Fig. 3A through C) and homology to IAV M1 suggests that DHOV M may contain NLS. Aberrant folding of truncated DHOV M fragments 1–136 and 103–170 could partially sequester NES1, making it unavailable for interaction with CRM1 and thus diminishing its cytoplasmic localization. Different levels of cytoplasmic localization from the same region of DHOV M (e.g., fragments 103–170 vs 103–136, Fig. 5B) suggest that fusion protein localization is partially dependent on surrounding sequences.

In IAV, vRNP export is highly sensitive to LMB treatment, with doses as little as 5 nM resulting in complete nuclear retention of IAV H1N1 (A/WSN/33) vRNPs in A549 cells (33, 49). This inhibitory concentration of LMB closely matches the LMB IC_50_ of 2.96 nM that we determined for DHOV in Huh7 cells (Fig. 1B). The sensitivity of DHOV to LMB suggests that CRM1 inhibition may be a viable therapeutic strategy for thogotovirus infections; however, the use of LMB *in vivo* is limited by its cytotoxicity (50). To address this limitation, derivative CRM1 inhibitors known as selective inhibitors of nuclear export (SINE) were developed to minimize cytotoxicity (51). One such compound, verdinexor, was shown to potently and selectively inhibit vRNP export in A549 cells infected with various IAV or IBV strains, with IC_50_ values ranging from 10 nM to 420 nM (52). Importantly, prophylactic and therapeutic treatment with verdinexor was also shown to reduce viral load and pathology of IAV infection while improving survival in a mouse model (52). These insights suggest that CRM1 inhibition by SINE compounds may be a potential therapy for DHOV and other thogotovirus infections, given the shared sensitivity of both DHOV and IAV to LMB treatment.

Overall, our study represents the first characterization of the nuclear export of progeny vRNPs of DHOV, a member of the *Thogotovirus* genus. We demonstrate that DHOV utilizes CRM1 as the primary exportin for vRNP egress, with M serving as an NEP by interacting with CRM1 via an NES. These findings enhance our understanding of thogotovirus biology and suggest that CRM1 inhibition may offer a potential therapeutic approach for treating thogotovirus infections.

## MATERIALS AND METHODS

### Alignment

To generate an alignment of multiple thogotovirus segment 6 ORFs, the amino acid sequence of the DHOV (strain I-611313; GenBank Accession: PQ469003- PQ469008), BRBV (strain Original; GenBank Accession: KP657750.3), OZV (strain EH8; GenBank Accession: NC_040734.1), Thailand tick thogotovirus (strain THOV/Boophilus sp./Thailand; GenBank Accession: NC_078597.1), Sinu virus (strain CoB 38d; GenBank Accession: NC_078772.1), THOV (strain IIA; GenBank Accession: AF527529.1), Upolu virus (GenBank Accession: NC_078650.1), and Jos virus (GenBank Accession: HM627172.1) M proteins were all aligned using CLUSTAL W (53).

### Antibodies

A custom affinity purified rabbit polyclonal antibody was produced by Biomatik against peptide Cys-^163^EDEQRDLWLEEVTRQLNTLTPVIRG^187^ from BRBV M and confirmed to cross-react to DHOV M prior to being used as a primary antibody. Rabbit monoclonal antibody against CRM1 was purchased from Cell Signaling Technology (46249S). Polyclonal mouse antibodies against DHOV NP were generated in-house. Briefly, BALB/c mice were immunized intramuscularly into the quadriceps with 25 µg of endotoxin-free pCAGGS-DHOV-NP combined with 25 µg of endotoxin-free plasmid expressing murine granulocyte-macrophage colony-stimulating factor (GMCSF) under the CMV promoter (pG-CMVi-GMCSF), as previously described (54–56). Mice were boosted with the same protocol twice at 3 week intervals. Four weeks after the final booster immunization, mice were anesthetized and exsanguinated, and whole blood samples were centrifuged at 13,000 × *g* for 10 minutes to separate out the serum. Whole serum was then utilized in place of the purified antibody for all subsequent immunofluorescence experiments. Mouse monoclonal antibody against the FLAG M2 epitope was purchased from Sigma-Aldrich (F3165). Goat anti-rabbit AlexaFluor 488 (ThermoFisher Scientific, A11008), goat anti-mouse AlexaFluor 594 (Invitrogen, A32740), or goat anti-mouse AlexaFluor 488 (Invitrogen, A11029) were used as secondary antibodies.

### Biosafety

All experiments using infectious DHOV were performed in biosafety level 2+ (BSL-2+) or biosafety level 3 (BSL-3) facilities at Mayo Clinic in accordance with approval and guidelines from the Mayo Clinic Institutional Biosafety Committee (IBC). Sample inactivation and removal from said facilities were performed in accordance with standard operating protocols approved by the IBC.

### Cell viability assay

Huh7 cells (1 × 10^4^ cells/well) were seeded in 96-well plates 1 day before compound treatment. Briefly, existing medium on cells was replaced with 2% FBS 1% P/S DMEM supplemented with indicated doses of LMB or vehicle control and incubated at 37°C for 1 hour. Following incubation, the compound-containing media was removed and replaced with 2% FBS 1% P/S DMEM before being incubated at 37°C for 24 hours. Cell viability was then assessed using the CellTiter Glo Luminescent Cell Viability Assay (Promega) according to the manufacturer’s instructions.

### Cells and virus

Huh7 (a kind gift from Yoshiharu Matsuura, Osaka University), HEK-293 (ATCC, CRL-1573), HEK-293T/17 (ATCC, CRL-11268), and Vero-E6 cells (ATCC, CRL-1586) were maintained in DMEM supplemented with 10% heat-inactivated fetal bovine serum (FBS; Thermo-Fisher Scientific) and 1% penicillin-streptomycin (P/S; Millipore Sigma) at 37°C in 5% CO_2_. An isolate of Dhori virus (DHOV) strain I-611313 was kindly provided by Brandy J. Russell of the Centers for Disease Control and Prevention (CDC). DHOV working stocks were prepared in Huh7 cells by passaging the original virus once and frozen at −80°C until use. DHOV stocks for sequencing were prepared in BHK-21 cells by passaging the original virus once.

The DHOV titer was determined by focus-forming assay. Briefly, Vero-E6 cells were seeded into 96-well plates (2 × 10^4^ cells/well) 1 day before infection. Cells were infected with 10-fold serial dilutions of the virus. After 1 hour absorption with tilting every 15 minutes, cells were overlayed with a mixture of 1.2% carboxymethylcellulose and Temin’s modified eagle medium (MEM). Two days post-infection, cells were fixed with 10% neutral buffered formalin before proceeding to immunofluorescence.

### Chemical compounds

Leptomycin B (L2913) was purchased from Millipore Sigma and stored at −20°C until use. The corresponding vehicle control was prepared by mixing molecular-grade methanol and water at a 7:3 ratio and stored at −20°C until use. Cycloheximide (01810) was purchased from Millipore Sigma, dissolved in sterile water, filter-sterilized, and stored −20°C until use.

### Colocalization analysis

Colocalization between indicated channels was quantified using the Just Another Colocalization plugin v2.1.4 (JaCoP) (57) in ImageJ (v1.54g) (58). If needed, thresholds were manually adjusted in JaCoP prior to quantification to appropriately cover the fluorescent signal.

### DHOV growth kinetics

Huh7 cells (3 × 10^6^ cells/well) were seeded into 6-well plates 1 day before infection. Cells were infected with DHOV at a multiplicity of infection (MOI) of 0.1. After 1 hour adsorption with tilting every 15 minutes, cells were washed three times with serum-free DMEM, and 3.0 mL of DMEM supplemented with 2% FBS was added to the cells. Immediately after adding the medium, 0.5 mL of supernatants was harvested as a sample of 0 dpi. Up to 3 dpi, 0.5 mL of supernatants was harvested at the indicated time points and replaced with an equal volume of fresh medium supplemented with 2% FBS. All supernatant samples were stored at −80°C until use for titration.

### CRM1, DHOV NP, and DHOV M localization in infected cells

Huh7 cells (0.5 × 10^5^ cells/well) were seeded in 24-well plates 1 day before infection and then infected with DHOV at an MOI of 100. Cells and virus were incubated for 1 hour at 37°C, with rocking every 15 minutes. The inoculum was then removed, and cells were washed three times with serum-free DMEM supplemented with 1% P/S. If undergoing compound treatment, cells were then treated with the indicated doses of compound in 2% FBS, 1% P/S DMEM for an additional hour at 37°C. Infected or compound-treated media was replaced with 2% FBS 1% P/S DMEM, and cells were incubated for a total of 2, 5, or 7 hours at 37°C following initial infection before proceeding to immunofluorescence. Rabbit anti-CRM1, mouse anti-DHOV NP, and rabbit anti-BRBV M were used as primary antibodies at a concentration of 1:500. Goat anti-rabbit AlexaFluor 488 and goat anti-mouse AlexaFluor 594 were used as secondary antibodies. Fluorescence images were obtained using a confocal microscope (ZEISS LSM 980 with Airyscan2) under 10 × magnification.

### Evaluation of LMB impact on DHOV titer

Huh7 cells (0.5 × 10^5^ cells/well) were seeded in 24-well plates 1 day before infection and then infected with DHOV at an MOI of 0.1. Cells and viruses were incubated for 1 hour at 37°C, with rocking every 15 minutes. The inoculum was then removed, and cells were washed three times with serum-free DMEM supplemented with 1% P/S. Subsequently, 0.5 mL of 2% FBS 1% P/S DMEM with the indicated concentration of LMB or vehicle control was added. Following a 1 hour incubation at 37°C, compound-containing media was replaced with 2% FBS 1% P/S DMEM, and cells were incubated for 24 hours at 37°C. Supernatants were then harvested and stored at −80°C until use for titration.

### Identification of potential nuclear export sequences

Potential NES were identified by uploading the DHOV M ORF into LocNES (40).

### Immunofluorescence assays

Cells were fixed in 10% neutral buffered formalin, permeabilized using a 1:1 ratio of methanol:acetone for 10 minutes at room temperature and blocked in 1% bovine serum albumin (BSA) in PBS for at least 1 hour at room temperature. Primary antibodies were added at a concentration of 1:1,000 (unless otherwise noted) in PBS containing 1% BSA and incubated overnight at 4°C with rocking. Secondary antibodies were added at concentrations of 1:10,000 in PBS and incubated at room temperature for 1 hour with rocking. Nuclei were stained using Hoechst 33342 (ThermoFisher Scientific) according to the manufacturer’s instructions.

### Mammalian two-hybrid assay

HEK-293 (0.8 × 10^5^ cells/well) were seeded into 24-well plates 1 day before transfection. Cells were then co-transfected with 100 ng of the indicated pBIND and pACT fusion constructs and 100 ng pG5*luc* per well using *Trans*IT-LTI (Mirus Bio) according to the manufacturer’s instructions. After 96 hours of incubation post-transfection, cells were harvested and lysed with passive lysis buffer (Promega). Luciferase assays were performed using a dual-luciferase reporter assay system (Promega) according to the manufacturer’s instructions. *Renilla* luciferase activity was used to normalize data to transfection efficiency using the following equation: (Firefly/*Renilla*). If needed, data were further normalized to corresponding vector controls: (sample/vector).

### Plasmid generation

Human RNA polymerase I (hPolI)-driven DHOV reverse genetics plasmids were generated for each segment. Briefly, DHOV segments 1–6 were amplified by RT-PCR from viral genomic RNA using segment-specific RT primers. cDNA was amplified using primers specific to the 3′ and 5′ NCR of each segment and cloned into a pPolI vector containing the hPolI promoter and terminator sequences as previously described (59, 60). To generate expression vectors of each DHOV protein, the PB2, PB1, PA, GP, NP, and M ORFs were amplified, and the cDNA was cloned into a pCAGGS vector that contains the cytomegalovirus enhancer fused to the chicken beta-actin promoter, as previously described (60, 61). FLAG-tagged expression vectors of each DHOV protein were created by amplifying each DHOV ORF with primers that inserted a FLAG tag epitope at either the N-terminus directly after the start codon or the C-terminus directly before the stop codon. These FLAG-tagged ORFs were then cloned back into the pCAGGS expression vector.

Plasmids pACT-Vector, pBIND-Vector, pACT-MyoD, pBIND-ID, and pG5*luc* were obtained from the CheckMate Mammalian Two-Hybrid System from Promega (E2440) (37). The human CRM1 ORF was amplified from pHAGE-XPO1—a gift from Gordon Mills & Kenneth Scott (Addgene plasmid #116804; http://n2t.net/addgene:116804; RRID:Addgene_116804)—and then inserted into the pACT and pBIND vectors (62). The IAV NEP ORF was amplified in fragments to remove the NS1 coding region. Briefly, NEP nucleotides 24–221 were amplified from an expression vector of the IAV (A/WSN/1933(H1N1)) NS1 ORF (NS1 nucleotides 496–693) using a primer that also contained NEP nucleotides 1–23. NEP nucleotides 222–366 were ordered as two overlapping oligos. Overlapping extension PCR was then utilized to combine the three fragments into the NEP ORF before cDNA was inserted into the pACT and pBIND vectors. To generate pACT and pBIND constructs of DHOV NP and M, the NP and M ORFs were amplified from the untagged pCAGGS constructs and inserted into the vector backbones. Successive DHOV M fusions to mCherry were generated by amplifying the indicated section of DHOV M from pCAGGS-DHOV-M and then performing overlapping extension PCR to fuse the fragment to the C-terminus of mCherry directly before the stop codon. These fusion protein constructs were then inserted back into the pCAGGS vector. Point mutations in the DHOV M NES sequence were generated by PCR mutagenesis on the DHOV M ORF prior to it being reinserted into the pCAGGS, pACT, pBIND, and pPolI vectors.

### Plaque analysis

Images of 30 separate plaques from titration plates of the indicated viruses were taken using a confocal microscope (ZEISS LSM 980 with Airyscan2) under 10× magnification. Individual images were uploaded into ImageJ (v1.54g) (58), and a line was drawn border to border over the widest section of each individual plaque to calculate plaque diameter in pixels. The area of each plaque in pixels was determined by manually tracing its border using the freehand tool in ImageJ. Pixel measurements were subsequently converted to micrometers (µm) using the width of the scale bar included in the image as a reference.

### Rescue of rDHOV

HEK-293T cells (2 × 10^5^ cells/well) were seeded into 12-well plates 1 day before transfection. Cells were co-transfected with 50 ng of each helper plasmid (pCAGGS-DHOV-PB2, pCAGGS-DHOV-PB1, pCAGGS-DHOV-PA, and pCAGGS-DHOV-NP) and 125 ng of each viral genome plasmid encoding segments 1–5 (pPolI-DHOV-S1, pPolI-DHOV-S2, pPolI-DHOV-S3, pPolI-DHOV-S4, and pPolI-DHOV-S5) and one version of segment 6 (pPolI-DHOV-S6-WT, -All A, -All G, -All R, −121/2 A or −121/2 S) using *Trans*IT-LTI (Mirus Bio) according to the manufacturer’s instructions. For the S1(-) and PB2(-) controls, pPolI-DHOV-S1 or pCAGGS-DHOV-PB2 were substituted for an equivalent amount of the empty vector control (pPolI-Empty or pCAGGS-Empty, respectively). After 24 hours of incubation, the cellular supernatant was removed and replaced with 1 mL of 2% FBS 1% P/S DMEM. Forty-eight hours post media change, cellular supernatant was harvested and stored at −80°C until use for titration.

### Single protein localization in transfected cells

All transfections were performed using *Trans*IT-LTI (Mirus Bio) according to the manufacturer’s instructions. To assess DHOV protein localization, Huh7 cells were seeded in 12-well plates 1 day before transfection with 1 ug of the indicated pCAGGS expression vector. After 24 hours, cells were directly fixed or treated with 100 nM LMB, vehicle control, or untreated media for 1 hour and then fixed. Protein localization was assessed using mouse anti-FLAG M2 or rabbit anti-BRBV M (1:500) as primary antibodies and goat anti-rabbit AlexaFluor 488 or goat anti-mouse AlexaFluor 488 as secondary antibodies. Fluorescence images were obtained using a ZOE Fluorescent Cell Imager (Bio-Rad) under 20 × magnification or a confocal microscope (ZEISS LSM 980 with Airyscan2) under 10 × magnification.

To assess mCherry fusion protein localization, HEK-293 cells (1 × 10^5^ cells/well) were seeded into 12-well plates 1 day before transfection with 0.5 µg of the indicated pCAGGS expression vector. To evaluate localization following compound treatment, cells were prepared as described above and 24 hours post-transfection were treated for 2 hours with 2% FBS 1% P/S DMEM containing 100 µg/µL cycloheximide and 100 nM LMB, vehicle control, or untreated media before fixation. Fluorescence images were obtained using a Nikon Eclipse Ts2 microscope with an Excelis MPX-6 camera under 20 × magnification.

### Statistical analysis

Experiments were conducted with at least three independent replicates. Statistical analysis was performed using the *t*-test and one-way ANOVA with GraphPad Prism 10 version 10.3.0.

### Viral genome sequencing

To sequence the complete genome of DHOV-I-611313, viral RNA was extracted from DHOV stocks from infected BHK-21 cells and purified using the QIAamp Viral RNA Mini kit (QIAGEN). Full-length viral genome sequences were determined using 3′ and 5′ RACE and Sanger sequencing.

## Data Availability

All DHOV sequences were deposited in the GenBank database under accession numbers PQ469003 to PQ469008.
